# Combined analysis of 16S rDNA sequencing and metabolomics to find biomarkers of drug-induced liver injury

**DOI:** 10.1038/s41598-023-42312-w

**Published:** 2023-09-13

**Authors:** Kaini He, Mimi Liu, Qian Wang, Sijie Chen, Xiaoyan Guo

**Affiliations:** Department of Gastroenterology, The Second Affiliated Hospital of Xi’an Jiaotong, University, Xi’an, Shaanxi China

**Keywords:** Microbiology, Biomarkers, Hepatitis

## Abstract

Drug induced liver injury (DILI) is a kind of liver dysfunction which caused by drugs, and gut microbiota could affect liver injury. However, the relationship between gut microbiota and its metabolites in DILI patients is not clear. The total gut microbiota DNA was extracted from 28 DILI patient and 28 healthy control volunteers (HC) and 16S rDNA gene were amplified. Next, differentially metabolites were screened. Finally, the correlations between the diagnostic strains and differentially metabolites were studied.The richness and uniformity of the bacterial communities decreased in DILI patients, and the structure of gut microbiota changed obviously. *Enterococcus* and *Veillonella* which belong to Firmicutes increased in DILI, and *Blautia* and *Ralstonia* which belong to Firmicutes, *Dialister* which belongs to Proteobacteria increased in HC. In addition, these diagnostic OTUs of DILI were associated with the DILI damage mechanism. On the other hands, there were 66 differentially metabolites between DILI and HC samples, and these metabolites were mainly enriched in pyrimidine metabolism and steroid hormone biosynthesis pathways. Furthermore, the collinear network map of the key microbiota-metabolites were constructed and the results indicated that Cortodoxone, Prostaglandin I1, Bioyclo Prostaglandin E2 and Anacardic acid were positively correlated with *Blautia* and *Ralstonia*, and negatively correlated with *Veillonella*.This study analyzed the changes of DILI from the perspective of gut microbiota and metabolites. Key strains and differentially metabolites of DILI were screened and the correlations between them were studied. This study further illustrated the mechanism of DILI.

## Introduction

The digestive tract of a healthy adult contains about 10^14^ bacteria, which can weigh about 1–2 kg, accounting for about 78% of all the bacteria in the human body^1^. The structural changes of gut microbiota and liver disease interact each other. Current studies have found that gut micro-environment disorder is closely related to a variety of liver diseases, including viral hepatitis, alcoholic hepatitis, non-alcoholic fatty liver disease, autoimmune hepatitis, cirrhosis and etc.^2–8^

Gut microbiota plays important roles in the pathogenesis of liver diseases. The disorder of gut microbiota have harmful effects on the host through various ways^9^. Gut microbiota could influence drug metabolism directly or indirectly. The essential drug bio-transformation processes in gut microbiota include reduction and hydrolysis reactions, demethylation, deamination, dehydroxylation, deacylation, decarbonylation and oxidation^10^. Gut microbiota could affect the process of drug metabolism, human susceptibility to DILI, innate immune system and gene expression involved in drug metabolism^10–12^.

In our study, the total fecal microbiota DNA was extracted and the full-length of 16S rDNA gene of DILI patient and healthy control volunteers (HC) were amplified. Alpha-diversity, Beta-diversity and species composition diversity were analyzed to explore the changes of microbiota in DILI. Then, the function of the diagnostic strains and differentially metabolites were analyzed. Finally, the correlation analysis between microbiota and metabolites was performed to further understand the mechanism of key strains in DILI.

## Materials and methods

### Data extraction

Fecal samples were provided by 28 DILI patients and 28 healthy control volunteers (HC) from The Second affiliated Hospital of Xi'an Jiaotong University for the last two years. In department of gastroenterology, we selected inpatients who met diagnostic criteria of DILI and excluded inpatients who used antibiotics or microecological preparations in the last 3 months or had other severe diseases, like liver cancer or other gastrointestinal disorders. The total fecal microbiota DNA was extracted and full length sequence of 16S rDNA were amplified by polymerase chain reaction (PCR). Agarose gel electrophoresis (AGE) was used to detect the amplification products. Gut microbiota composition was evaluated using PacBio sequencing. The sequences were binned into operational taxonomic units (OTUs) based on 97% identification by Usearch. All participants signed informed consent forms. This study has passed the review of The Second affiliated Hospital of Xi'an Jiaotong University Ethics Committee.

### Species diversity analysis

The relative abundance curve was used to reflect species diversity and abundance of samples. Alpha-diversity indexes, including Chao1, ACE, observed species and goods Coverage were compared to analysis the species richness and uniformity^13^. Beta-diversity analysis was used to evaluate the species complexity among different groups. In this study, principal co-ordinates analysis (PCoA) was used to show the differences in the different groups. Then, the analysis of similarities (Anosim) was used to analyze whether the differences between two groups were significantly greater than that within the groups^14^.

### Analysis of the species composition diversity

The differences of gut microbiota composition were further analyzed. The distribution of gut microbiota at the phylum and genus level in different groups were compared, and the characteristic strains were researched by Linear discriminant analysis Effect Size (LEfSE), OTUs with relative abundance greater than 0.5% were screened for analysis based on Linear Discriminant Analysis (LDA) score (http://huttenhower.sph.harvard.edu/lefse/)^15^.

### Functional prediction for the diagnostic OTUs

The receiver operating characteristic (ROC) curves were calculated by “pROC” R package (version 1.18.0), and the area under the ROC curve (AUC) value was used to access the distinguish ability of characteristic strains^16^. The functional prediction of the diagnostic strains (AUC > 0.6) were analyzed and the differences between DILI patients and HC were studied by “PICRUSt2” R package (version 0.2.3)^17^.

### Differentially analysis of the metabolites

The metabolic differences between DILI patient and HC were showed by constructing the partial least-squares-discriminant analysis (PLS-DA) model, and the model was assessed by permutation test. Then, the metabolites were selected using three methods, including multiple change method (FC value), T test method (P value, FDR value), and PLS-DA method (VIP value). Differentially metabolites between this two groups were screened with the conditions of VIP > 1, FC ≥ 2, and *p* < 0.01. The “ggplot2” R package (version 3.3.3) and the “pheatmap” R package (version 1.0.12) were used to draw the volcano map and heat map, respectively^18, 19^. In addition, the enrichment analysis of differentially metabolites were analyzed by online website “MetaboAnalyst” (https://www.metaboanalyst.ca)^20^.

### Correlation analysis of the microbiota and the metabolites

The correlations between the diagnostic strains and differentially metabolites were studied by “Spearman” (|r| ≥ 0.3, *p* < 0.05). Metabolites in the pair were regarded as key metabolites and diagnostic strains were regarded as key microbiota. Besides, the ROC curves for the key metabolites were used to access the distinguish ability. Finally, the collinear network map of the key microbiota-metabolites were constructed by “Cytoscape” (version 3.7.1)^21^.

### Ethics approval and consent to participate

This study was performed in accordance with the Declaration of Helsinki and was approved by the medical ethics committee of the Second Affiliated Hospital of Xi'an Jiaotong University (2015162). All participants provided their written informed consent to participate in this study.

## Results

### Species diversity analysis

**Figure 1:**
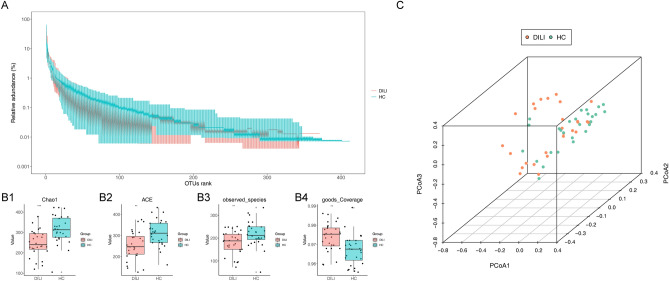
Species diversity analysis. (**A**) Relative abundance curve of gut microbiota. Abscissa: OTU rank, representing OTU arranged according to abundance in the sample; Ordinate: The relative abundance of the number of sequences in this rank OTU is the number of sequences belonging to the OTU divided by the total number of sequences, green is HC, red is DILI. (**B**) Alpha-diversity indexes. (**B1**) Chao1 index; (**B2**) ACE index; (**B3**) observed species index; (**B4**) goods Coverage index.Abscissa: different groups, green is HC, red is DILI; Ordinate: diversity index value, where the upper left corner is p value display, *p* < 0.001: ***, *p* < 0.01: **, *p* ≤ 0.05: *(**C**) PCoA analysis. HC is shown in green and DILI in red, representing samples from different environments or conditions. The horizontal and vertical axes are relative distances and have no practical significance.

In this experiment, the relative abundance curve was balanced as the number of reads increases, which indicated that enough sequencing data were obtained and the sequencing depth were reasonable (Fig. 1A).

Alpha-diversity indexes of each group were shown in Fig. 1B. The Chao1 and ACE index of DILI groups were significantly lower than HC groups, which indicated that the richness of bacterial community decreased obviously in DILI patients (*p* < 0.05). On the other hands, the observed species index of DILI groups was significantly lower than HC groups, and the goods Coverage index of DILI groups was significantly higher than HC groups, which indicated that the uniformity of the bacterial communities decreased obviously in DILI patients.

The PCoA results showed that DILI patient and HC clustered separately (Fig. 1C). Besides, greater differences between groups than within-group (ANOSIM statistics = 0.0868, *p* = 0.01) indicated that grouping was meaningful and the structure and composition of gut microbiota were significantly changed by DILI.

### Analysis of the species composition diversity

**Figure 2:**
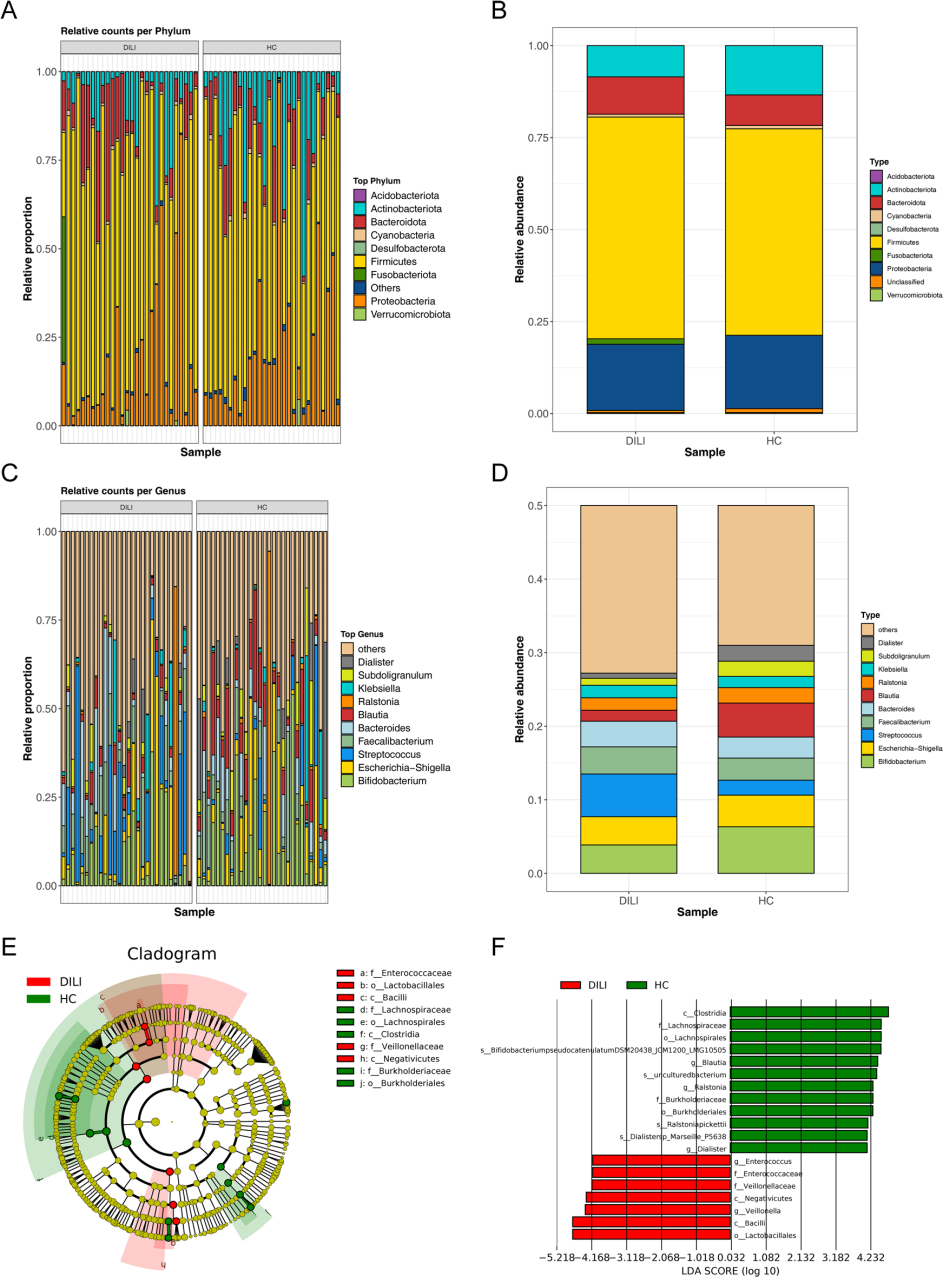
Analysis of the species composition diversity. (**A**) Relative proportion at the phylum in samples. The abscissa is samples of DILI and HC groups, the ordinate is the relative abundance, and different colors represent different phylum. (**B**) Different abundance of microbiota at the phylum between DILI and HC groups. The abscissa is the group, the ordinate is the relative abundance, and different colors represent different phylum. (**C**) Relative proportion at the genus in samples. The abscissa is samples of DILI and HC groups, the ordinate is the relative abundance, and different colors represent different genus. (**D**) Different abundance of microbiota at the genus between DILI and HC groups. The abscissa is the group, the ordinate is the relative abundance, and different colors represent different genus. (**E**) Cluster trees of LEfSE analysis. Red and green areas represent different samples. In the tree, the red nodes represent the important microbiota in DILI, the green nodes represent the microbial groups that played an important role in the HC group, and the yellow nodes are the microbial groups that did not play an important role in the two groups. In the legend on the right, the English letters in the figure are the species names. The circles from inside to outside in the branching diagram are the classification level from phylum to species. The diameter of each small circle is proportional to the relative abundance in the gut microbiota, and the letters p, c, o, f, g and s represent phylum, class, order, family, genus and species, respectively. (**F**) Histogram of LDA analysis. LDA score was used to screen the differential microbiota. The microbial taxa with significant effects in the two samples are LDA score on the abscissa and differential microbiota on the ordinate.

The distribution of gut microbiota at the phylum and genus level in different groups were further analyzed. At the phylum, the gut microbiota were mainly composed of Firmicutes, Proteobacteria, Bacteroidetes and Actinobacteria (Fig. 2A). Compared with the HC groups, the abundance of Bacteroidota, Firmicutes, Fusobacteriota and Acidobacteriota in DILI groups were significantly increased, and the abundance of Proteobacteria, Verrucomicrobiota and Desulfobacterota were significantly decreased (Fig. 2B). At the genus, the gut microbiota were mainly composed of *Bifidobacterium*, *Escherichia-Shigella*, *Streptococcus*, *Faecalibacterium*, *Bacteroides*, *Blautia*, *Ralstonia*, *Klebsiella*, *Subdoligranulum* and *Dialister* (Fig. 2C). The abundance of *Streptococcus*, *Faecalibacterium*, *Bacteroides* and *Klebsiella* were significantly increased, while the abundance of *Bifidobacterium*, *Blautia*, *Subdoligranulum* and *Dialister* were significantly decreased in DILI groups (Fig. 2D). To research the key gut strains, the LEfSE analysis was further conducted and differential abundance of 19 gut strains were obtained. As showed in Fig. 2E and F, *Enterococcus* and *Veillonella* which belong to Firmicutes significantly increased in DILI. *Blautia* and *Ralstonia* which belong to Firmicutes, *Dialister* which belongs to Proteobacteria significantly increased in HC.

### Screening for the diagnostic OTUs of DILI, which associated with the DILI damage mechanism

**Figure 3:**
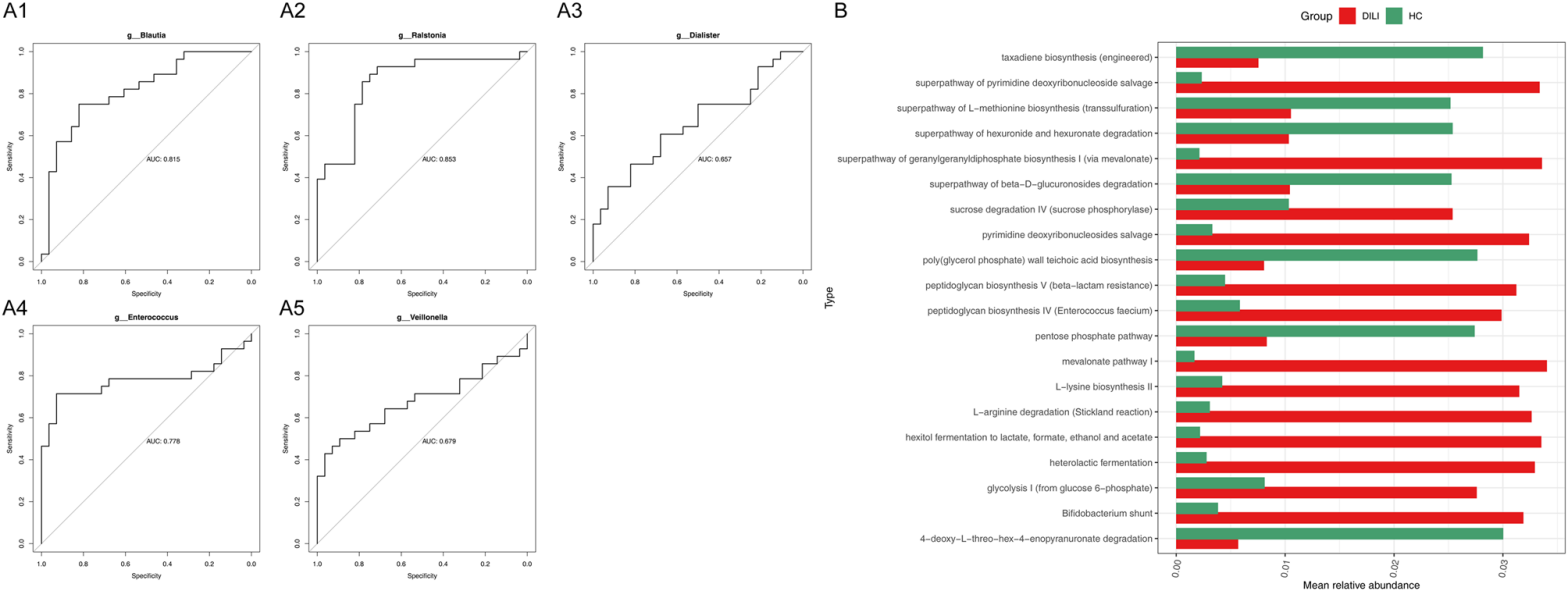
Analysis of the diagnostic OTUs of DILI. (**A**) The ROC curves of 5 characteristic strains (at the genus level). (**A1**) The ROC curves of *Blautia*; (**A2**) The ROC curves of *Ralstonia*; (**A3**) The ROC curves of *Dialister*; (**A4**) The ROC curves of *Enterococcus*; (**A5**) The ROC curves of *Veillohella*. (**B**) The functional prediction of the 19 diagnostic strains. The abscissa is the mean relative abundance of each group, and the ordinate is the pathway name.

In this study, the ROC curves of 19 characteristic strains involved were calculated and the AUC value were obtained between 0.65 and 0.9, where five out of characteristic gut microbiota at the genus level were displayed in Fig. 3A (AUC_*Enterococcus*_ = 0.778, AUC_*Veillohella*_ = 0.679, AUC_*Blautia*_ = 0.815, AUC_*Ralstonia*_ = 0.853, AUC_*Dialister*_ = 0.657). The rest of which was shown in Supplementary Fig. S1. In addition, the functional prediction of the 19 diagnostic strains were analyzed and the results showed that 219 pathways were significantly different between the two groups, among them, 4-deoxy-L-threo-hex-4-enopyranuronate degradation pathway and superpathway of L-methionine biosynthesis (transsulfuration) were significantly highly expressed in HC groups (*p* < 0.05), mevalonate pathway I were significantly highly expressed in DILI groups (*p* < 0.05) (Fig. 3B).

### The function of 66 differentially metabolites were mainly enriched in pyrimidine metabolism and steroid hormone biosynthesis pathways

**Figure 4:**
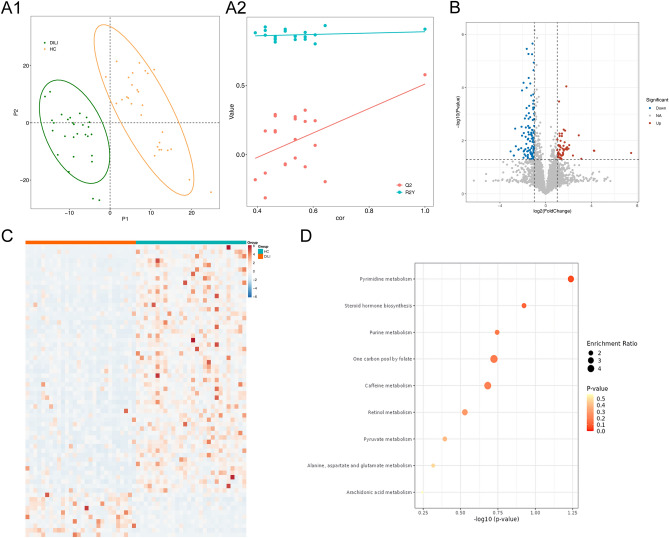
Analysis of the differentially metabolites. (**A**) The PLS-DA model. (**A1**) PLS-DA model. The abscissa and ordinate are the sample scores in the first two axes of PCA, that is, the sorting coordinates of each sample in PC1 and PC2 axes, according to which the differences in metabolite composition of each sample can be evaluated. Green is the DILI group, and orange is the HC group; (**A2**) Permutation test plot of PLS-DA model. The abscismal is the correlation coefficient between the original data and the replacement data, the ordinate is the R2Y value and Q2 value, the red point is the Q2 value, the green point is the R2Y value, the red line is the regression line of Q2, the green is the regression line of R2Y, the rightmost is the true value, and the left is the simulated value. (**B**) Volcano map of metabolites in samples. The abscission is log10 (*p* value), and the ordinate represents the log2(fold change). Each point in the figure represents a metabolite; red represents up-regulated differential genes, blue represents down-regulated differential metabolites, and gray represents non-significantly differential metabolites. (**C**) Heatmap of differentially metabolites.Each small square represents each differential metabolite, and its color indicates the amount of the differential metabolite. The higher the value, the darker the color (Red is high expression, and blue is low expression). (**D**) The functional prediction of different metabolites. The horizontal axis represents log10(*p* value) and the vertical axis represents enrichment pathways. Each dot in the figure represents an enrichment pathway, the lighter the color, the greater the *p* value, and the larger the dot, the greater the proportion of enrichment.

The PLS-DA model was constructed and the results showed that the composition of metabolites were different between these two groups (Q2 = 0.577, R2Y = 0.906) (Fig. 4A). Totals of 66 differentially metabolites, including 56 down-regulated and 10 up-regulated metabolites were screened in DILI groups under the VIP > 1, FC ≥ 2, *p* < 0.01 (Fig. 4B and C). In addition, these differentially metabolites were mainly enriched in pyrimidine metabolism and steroid hormone biosynthesis pathways (Fig. 4D).

### Correlation analysis of the gut microbiota and the metabolites

**Figure 5:**
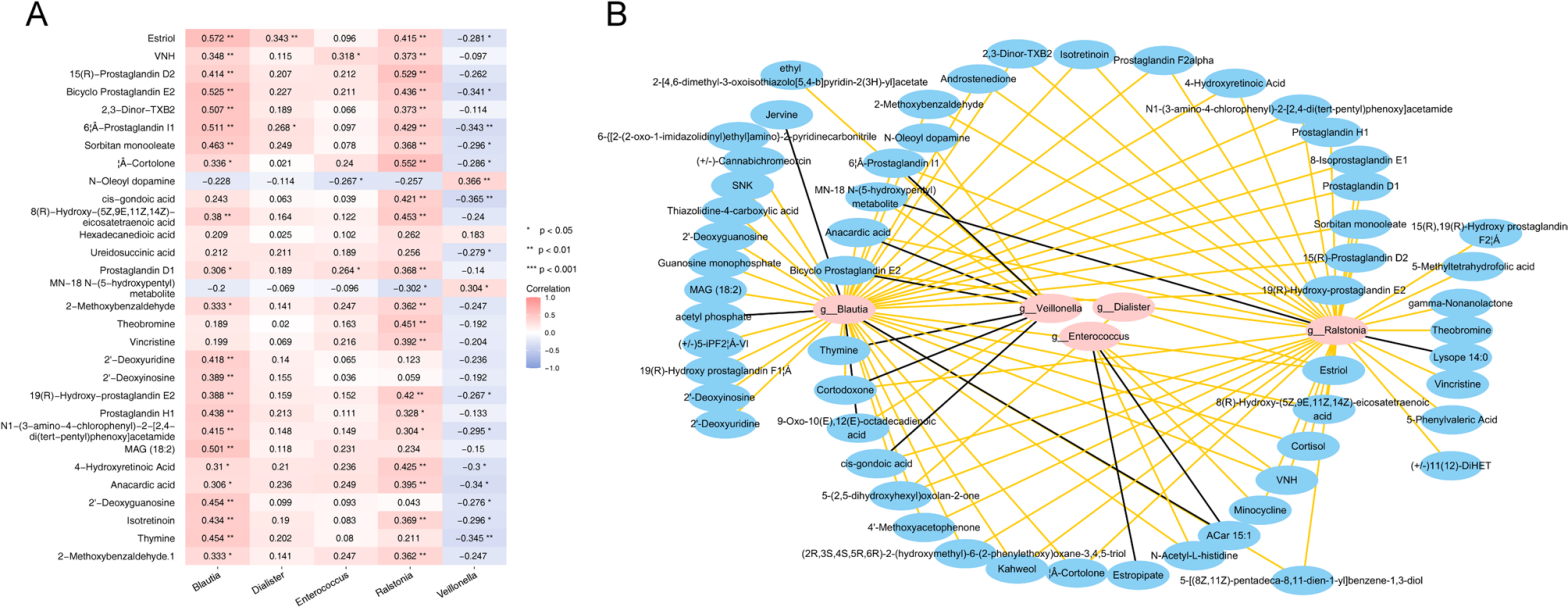
Correlation analysis of the microbiota and the metabolites. (**A**) The correlation between the differentially metabolites and diagnostic strains. The abscissa is the differential microbiota, the ordinate is the differential metabolites, each small cell represents the similarity, the more red color represents the stronger positive correlation, and the more blue color represents the stronger negative correlation. (**B**) The collinear network map of the key microbiota-metabolites. Pink dots are genus differential microbiota, blue dots are correlated differential metabolites, gray lines represent negative correlation, and yellow lines represent positive correlation.

The correlation between the differentially metabolites and five diagnostic strains at the genus level were showed in Fig. 5A. *Blautia*, *Dialister* and *Ralstonia* showed a strong correlation with the Estriol, and Cortolne, respectively, *Enterococcus* and *Veillonella* showed a strong correlation with the VNH and Oleoyl dopamine, respectively. The ROC curves for key metabolites suggested that most of key metabolites have diagnostic value, with the AUC value between 0.7 and 0.95 (Supplementary Fig. S2). Furthermore, the collinear network map of the key microbiota-metabolites was constructed and the results indicated that Cortodoxone, Prostaglandin I1, Bioyclo Prostaglandin E2 and Anacardic acid were positively correlated with *Blautia* and *Ralstonia*, and negatively correlated with *Veillonella* (Fig. 5B).

## Discussion

The human intestinal tract, especially the colon, is inhabited with a large number of microorganisms, they play central roles in the regulation of intestinal function, immune system maturation, pathogen defense, and substance metabolism^22^. Besides, gut microbiota are also associated with hepatic diseases. Current studies have found that gut microbiota disorder was closely related to liver diseases, including viral hepatitis, alcoholic hepatitis, non-alcoholic fatty liver disease, autoimmune hepatitis, cirrhosis and etc.^2–8^ Gut microorganisms metabolites and gut micribiota could act together on the liver, which could promote the occurrence and development of liver disease^23^. In vivo experiments indicated that gut microbiota and its metabolites were altered by DILI, and might involve in the process of liver injury^24–27^. However, the relationship among DILI, gut microbiota and metabolites are unclear, and the joint analysis between microbiome and metabolome have not been reported.

In our study, the richness and uniformity decreased obviously in DILI samples, and the species composition were different between 2 group samples. Significant changes in composition and structure of gut microbiota were confirmed in a variety of liver diseases. Zeng et al. have found that the alpha-diversity indexes of gut microbiota in patients with hepatitis B were higher than that in healthy human, and beta-diversity analysis showed significant differences between the two groups^28^. It has also been found that the alpha- and beta- diversity of gut microbiota were significantly altered in liver injury mouse caused by exposure to perfluorooctanoic acid (PFOA)^26^. Besides, a decrease in the diversity of gut microbiota has been found in mice with Acetaminophen (APAP) -induced liver injury^29^. Our findings are consistent with these results.

The gut bacterial strains associated with DILI identified by LEfSE analysis may be pathogenic agents of DILI and participate in the occurrence or development of DILI. These key strains may be potential biomarkers for the diagnosis or prediction of DILI on their own, or working in concert with other strains. In addition, certain strains may participate in the development of DILI as cofactors. Therefore, LEfSE analysis could help us learn more about the function and mechanisms of the strains about DILI. At the genus level, *Enterococcus*, *Veillonella* were significantly increased and *Blautia*, *Ralstonia*, *Dialister* were significantly decreased by DILI. Our results were similar to previous analyses. *Enterococcus* and *Veillonella* were significantly increased in Chronic viral hepatitis B^3, 28^. In autoimmune hepatitis, the abundance of *Veillonella* significantly increased, which is highly associated with primary biliary cirrhosis and primary sclerosing cholangitis^30^. Some studies found that the abundance of *Blautia* was further reduced in cirrhotic patients with hepatocellular carcinoma or HBV^31^. *Blautia* also decreased in HBV infected mice model, which may be related to the progression of liver injury^31, 32^. Few studies have shown the relationship between *Ralstonia* and liver injury. The abundance of *Dialister*, a beneficial bacterium, is significantly decreased in patients with chronic hepatitis B, and its decline is associated with the progression of liver injury in hepatitis B^3^. *Enterococcus* and *Veillonella* may be new targets for clinical diagnosis of drug-induced liver injury. In addition, in cirrhotic patients with no hepatocellular carcinoma, the abundance of *Blautia* and *Bifidobacterium* were increased, while *Enterococcaceae* and *Enterococcus* were decreased, which were different to our results^31^. These data showed that the gut bacterial strains were regulated by different liver injury types and disease status. Moreover, the relationship between key strains and DILI needs to be verified by further experiments in vivo and in vitro.

The functional prediction of the 19 diagnostic strains mentioned above showed that 4-deoxy-L-threo-hex-4-enopyranuronate degradation pathway and superpathway of L-methionine biosynthesis (transsulfuration) were significantly lowly expressed in DILI groups, and mevalonate pathway I were significantly highly expressed in DILI groups. The 4-deoxy-L-threo-hex-4-enopyranuronate degradation pathway, a kind of sugar derivative degradation, is involved in short chain fatty acids (SCFAs) production. Dietary soluble fibers are fermented by gut bacteria into SCFAs^33^. SCFAs are well known to exert a wide beneficial impact to the host^34, 35^. Glucuronate degradation is associated with hepatic drug metabolism^36^. SCFAs have been reported to be associated with liver metabolic diseases, including butyrate and valerate^37, 38^. Therefore, promoting the 4-deoxy-L-threo-hex-4-enopyranuronate degra dation pathway might be the key to the treatment of DILI. Mevalonate is associated with the mechanism of DILI^39, 40^. Previous studies showed that methionine and its metabolic enzyme levels were reduced in NAFLD, and the decreaced methionine plasma level and impairment of transsulfuration pathway in NAFLD might be related to the liver injury^41, 42^. S-adenosylmethionine is an effective chemopreventive agent in HCC and other cancers^43^. The decrease of superpathway of L-methionine biosynthesis might be associated with the liver injury. Furthermore, related studies have shown that isoprenoids, cholesterol (vital products of the mevalonate pathway) and HMG-COA (key enzymes of mevalonate pathway) were significantly associated with liver injury^44, 45^. Besides, Liu et al. showed that the importance of cholesterol/mevalonate pathway in the progress of NAFLD^46^. Therefore, the mevalonate pathway I may affect the process of DILI by producing isoprenoids and cholesterol et al. Bile acid (BA) metabolism is associated with the development of liver injury. The treatment of NAFLD by interfering with BA synthesis and metabolism has become a new research direction. But we didn’t find BA metabolism enriched pathway in our results^47^. These data showed that the BA metabolism was not the main process in development of DILI. Furthermore, the effect of gut metabolic products on DILI needs more studies to verify.

Previous study showed that pyrimidine metabolism is linked to liver injury, such as fatty liver and alcoholic liver disease^48, 49^. Sexual hormones and adrenocortical hormone are products of steroidhormone biosynthesis. Sexual hormones affect endogenous and exogenous metabolism in the liver and involve in the pathogenesis of functional and structural disorders of the liver, including the development of hepatic steatosis, fibrosis, and carcinogenesis^50^. Elevated levels of glucocorticoids play an important role in the pathogenesis of nonalcoholic fatty liver disease, which might caused by hepatic cholesterol synthesis increasing and utilization decreasing^51^. Numerous studies have demonstrated that cirrhosis and noncritical chronic liver disease are associated with relative adrenal insufficiency (RAI)^52–54^. RAI is the term given to inadequate production of cortisol relative to the severity of illness^52^.The pyrimidine metabolism and steroid hormone biosynthesis pathways of differentially metabolites maybe involved in the progress of DILI. Prostaglandins (PG), belonged to eicosanoids, formed via the cyclooxygenase pathway^55^. Eicosanoids implicate the regulation of hepatocyte proliferation, liver regeneration and liver injury and recovery^55^. PG in protecting liver to the hepatic ischemia and reperfusion damage has been extensively studied^56^. Prostaglandin E2 (PGE2) could retard CCl4-induced liver fibrosis in rats^55, 57, 58^. Anacardic acid could trigger the classic activation pathway in macrophages by phosphorylating mitogen-activated protein kinases, thereby activating innate immunity^59, 60^. Besides, anacardic acid has been confirmed to have antitumor effects in some preclinical models^61^. In our study, Cortodoxone, Prostaglandin I1, Bioyclo PGE2 and Anacardic acid were also positively correlated with *Blautia* and *Ralstonia*, and negatively correlated with *Veillonella*. This shows that our results are reliable.

There are some potential limitations with this study. The study’s sample size was not large enough, even though the results were statistically significant. The conclusion of the study does not connect to clinical information. The results of this study showed the correlation among DILI, gut bacterial strains and metabolic products, but the results didn’t tell the causal relationship among them. We will collect more samples of DILI patients for further research through effective measures such as setting up a registration database and establishing communication with patients. In addition, the effect of clinical information such as age, sex, medical history, and disease status on key strains and differential metabolic products need further studies. We will perform experiments in vivo and in vitro to verify the function of gut bacterial strains and metabolic products in DILI, and figure out the causal relationship among them to promote our knowledge and understanding of DILI.

In conclusion, this study analyzed the changes of DILI from the perspective of gut microbiota and metabolites. The richness and uniformity of the bacterial communities decreased in DILI patients, and the structure of gut microbiota changed obviously. Key strains and differentially metabolites of DILI were screened and the correlations between the them were studied. *Enterococcus* and *Veillonella* which belong to Firmicutes increased, *Blautia* and *Ralstonia* which belong to Firmicutes, *Dialister* which belongs to Proteobacteria decreased in DILI. Differentially metabolites between DILI and HC samples were mainly enriched in pyrimidine metabolism and steroid hormone biosynthesis pathways. Furthermore, the Cortodoxone, Prostaglandin I1, Bioyclo Prostaglandin E2 and Anacardic acid were positively correlated with *Blautia* and *Ralstonia*, and negatively correlated with *Veillonella*. This study further illustrated the role of gut microbiota and metabolites in progress of DILI. We will continue focus on the mechanism of gut microbiota and its metabolites in DILI.

### Supplementary Information

Supplementary Figures.

## Data Availability

All data and material are available upon request to correspondence author.
