# Supplementary material for: Combined analysis of 16S rDNA sequencing and metabolomics to find biomarkers of drug-induced liver injury
Source: Sci Rep. 2023 Sep 13;13:15138. doi: 10.1038/s41598-023-42312-w (PMC10499917; doi:10.1038/s41598-023-42312-w)
Supplement: Supplementary file 1 — Supplementary Figures. [file 41598_2023_42312_MOESM1_ESM.pdf]

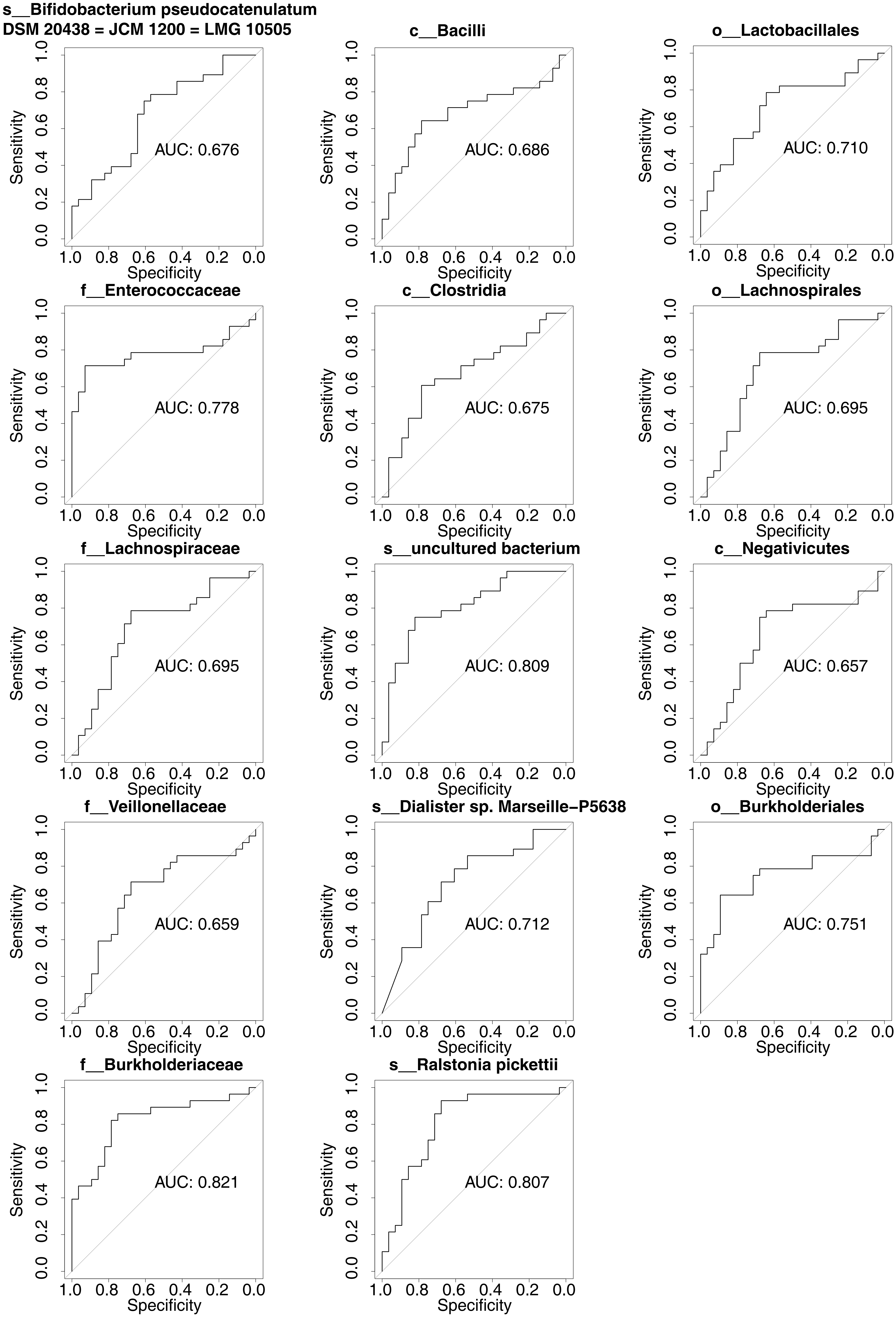
s__Bifidobacterium pseudocatenulatum DSM 20438 = JCM 1200 = LMG 10505
AUC: 0.676
c__Bacilli
AUC: 0.686
o__Lactobacillales
AUC: 0.710
f__Enterococcaceae
AUC: 0.778
c__Clostridia
AUC: 0.675
o__Lachnospirales
AUC: 0.695
f__Lachnospiraceae
AUC: 0.695
s__uncultured bacterium
AUC: 0.809
c__Negativicutes
AUC: 0.657
f__Veillonellaceae
AUC: 0.659
s__Dialister sp. Marseille-P5638
AUC: 0.712
o__Burkholderiales
AUC: 0.751
f__Burkholderiaceae
AUC: 0.821
s__Ralstonia pickettii
AUC: 0.807
Sensitivity
Specificity

Supplementary Fig. S1. The ROC curves of the resting 15 characteristic strains for predicting DILI.

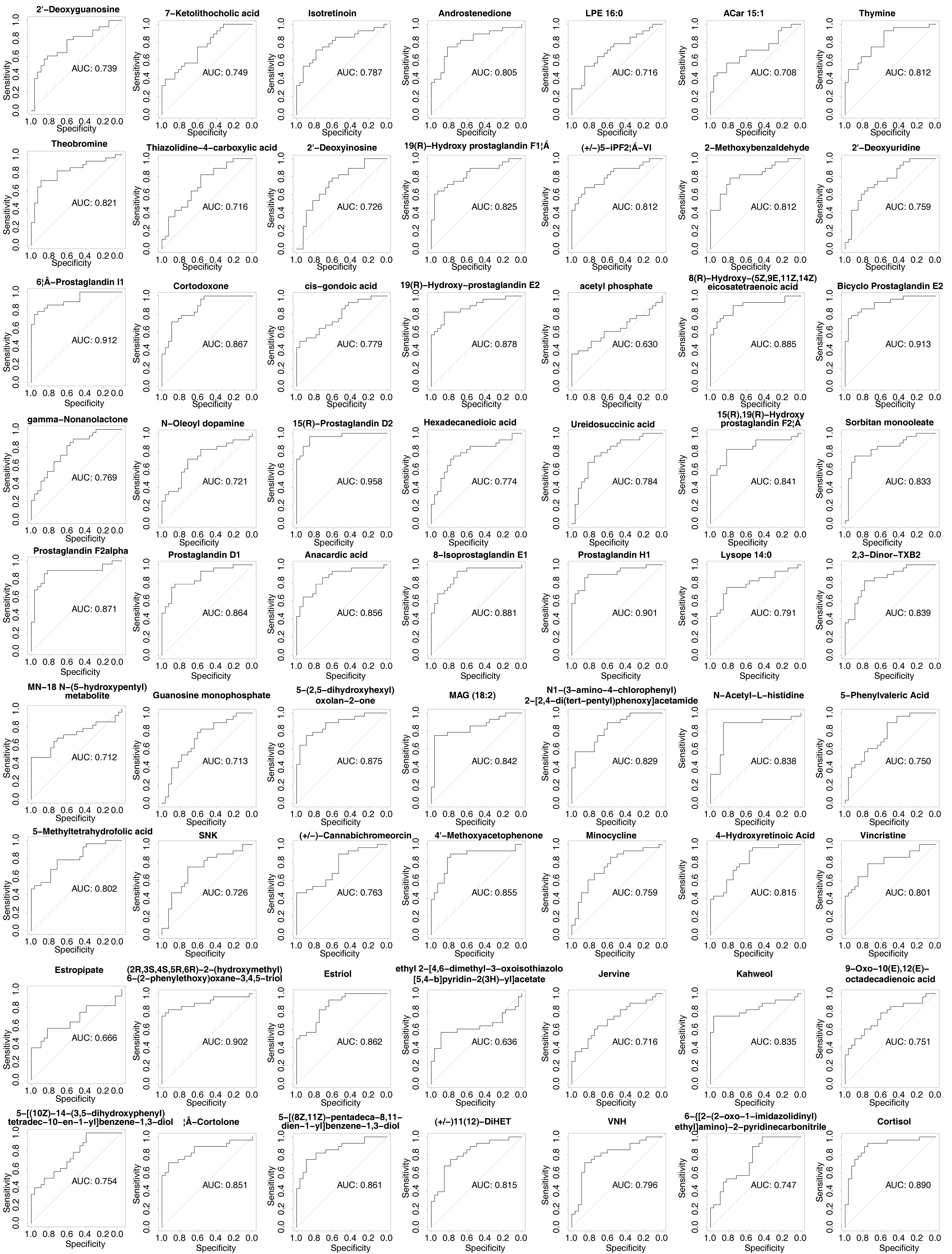
2'−Deoxyguanosine
AUC: 0.739
7−Ketolithocholic acid
AUC: 0.749
Isotretinoin
AUC: 0.787
Androstenedione
AUC: 0.805
LPE 16:0
AUC: 0.716
ACar 15:1
AUC: 0.708
Thymine
AUC: 0.812
Theobromine
AUC: 0.821
Thiazolidine−4−carboxylic acid
AUC: 0.716
2'−Deoxyinosine
AUC: 0.726
19(R)−Hydroxy prostaglandin F1¦Á
AUC: 0.825
(+/−)5−iPF2¦Á−VI
AUC: 0.812
2−Methoxybenzaldehyde
AUC: 0.812
2'−Deoxyuridine
AUC: 0.759
6¦Â−Prostaglandin I1
AUC: 0.912
Cortodoxone
AUC: 0.867
cis−gondoic acid
AUC: 0.779
19(R)−Hydroxy−prostaglandin E2
AUC: 0.878
acetyl phosphate
AUC: 0.630
8(R)−Hydroxy−(5Z,9E,11Z,14Z)
eicosatetraenoic acid
AUC: 0.885
Bicyclo Prostaglandin E2
AUC: 0.913
gamma−Nonanolactone
AUC: 0.769
N−Oleoyl dopamine
AUC: 0.721
15(R)−Prostaglandin D2
AUC: 0.958
Hexadecanedioic acid
AUC: 0.774
Ureidosuccinic acid
AUC: 0.784
15(R),19(R)−Hydroxy
prostaglandin F2¦Á
AUC: 0.841
Sorbitan monooleate
AUC: 0.833
Prostaglandin F2alpha
AUC: 0.871
Prostaglandin D1
AUC: 0.864
Anacardic acid
AUC: 0.856
8−Isoprostaglandin E1
AUC: 0.881
Prostaglandin H1
AUC: 0.901
Lysope 14:0
AUC: 0.791
2,3−Dinor−TXB2
AUC: 0.839
MN−18 N−(5−hydroxypentyl)
metabolite
AUC: 0.712
Guanosine monophosphate
AUC: 0.713
5−(2,5−dihydroxyhexyl)
oxolan−2−one
AUC: 0.875
MAG (18:2)
AUC: 0.842
N1−(3−amino−4−chlorophenyl)
2−[2,4−di(tert−pentyl)phenoxy]acetamide
AUC: 0.829
N−Acetyl−L−histidine
AUC: 0.838
5−Phenylvaleric Acid
AUC: 0.750
5−Methyltetrahydrofolic acid
AUC: 0.802
SNK
AUC: 0.726
(+/−)−Cannabichromeorcin
AUC: 0.763
4'−Methoxyacetophenone
AUC: 0.855
Minocycline
AUC: 0.759
4−Hydroxyretinoic Acid
AUC: 0.815
Vincristine
AUC: 0.801
Estropipate
AUC: 0.666
(2R,3S,4S,5R,6R)−2−(hydroxymethyl)
6−(2−phenylethoxy)oxane−3,4,5−triol
AUC: 0.902
Estriol
AUC: 0.862
ethyl 2−[4,6−dimethyl−3−oxoisothiazolo
[5,4−b]pyridin−2(3H)−yl]acetate
AUC: 0.636
Jervine
AUC: 0.716
Kahweol
AUC: 0.835
9−Oxo−10(E),12(E)−
octadecadienoic acid
AUC: 0.751
5−[(10Z)−14−(3,5−dihydroxyphenyl)
tetradec−10−en−1−yl]benzene−1,3−diol
AUC: 0.754
¦Â−Cortolone
AUC: 0.851
5−[(8Z,11Z)−pentadeca−8,11−
dien−1−yl]benzene−1,3−diol
AUC: 0.861
(+/−)11(12)−DiHET
AUC: 0.815
VNH
AUC: 0.796
6−{[2−(2−oxo−1−imidazolidinyl)
ethyl]amino}−2−pyridinecarbonitrile
AUC: 0.747
Cortisol
AUC: 0.890

Supplementary Fig. S2. The ROC curves for 63 key metabolites for predicting DILI.
